# Correction: The landscape of m1A modification and its posttranscriptional regulatory functions in primary neurons

**DOI:** 10.7554/eLife.91312

**Published:** 2023-07-25

**Authors:** Chi Zhang, Xianfu Yi, Mengfan Hou, Qingyang Li, Xueying Li, Lu Lu, Enlin Qi, Mingxin Wu, Lin Qi, Huan Jian, Zhangyang Qi, Yigang Lv, Xiaohong Kong, Mingjun Bi, Shiqing Feng, Hengxing Zhou

**Keywords:** Mouse

Zhang C, Yi X, Hou M, Li Q, Li X, Lu L, Qi E, Wu M, Qi L, Jian H, Qi Z, Lv Y, Kong X, Bi M, Feng S, Zhou H. 2023. The landscape of m1A modification and its posttranscriptional regulatory functions in primary neurons. *eLife* **12**:e85324. doi: 10.7554/eLife.85324.

Published 7 March 2023

After publication, both ourselves and attentive readers have identified an error in the **Materials and Methods** section of our article regarding the *Preparation of the m1A RNA immunoprecipitation sequencing (MeRIP-seq) library*. We rectified this error during the proofreading stage; however, due to potential network issues or other unknown reasons, our amendments were not reflected in the online version of the article. We sincerely apologize for this oversight and have now made the necessary corrections to the manuscript. It is important to note that these corrections do not alter the results or impact the scientific conclusions drawn in the original report. All authors have expressed their unanimous agreement with the correction text.

Corrected text:

GenSeq m1A MeRIP Kit (Cloudseq Biotech Inc, Shanghai, China; Cat. No. GS-ET-002) was used to perform immunoprecipitation (IP) of m1A RNA according to the manufacturer’s recommendations. IP buffer, Protein A/G beads, and m1A antibodies were used to prepare the magnetic beads for IP.

Original text:

A Magna MeRIP m1A Kit (Merck Millipore, MA, USA; Cat. No. 17–10499) was used to perform immunoprecipitation (IP) of m1A RNA according to the manufacturer’s recommendations. IP buffer (Cat. No. CS220009), Magna ChIP Protein A/G Magnetic Beads (Cat. No. CS203152), and an anti-m1A antibody (Cat. No. MABE1006) were used to prepare the magnetic beads for IP.

The article has been corrected accordingly.

