# Supplementary material for: The landscape of m1A modification and its posttranscriptional regulatory functions in primary neurons
Source: eLife. 2023 Mar 7;12:e85324. doi: 10.7554/eLife.85324 (PMC9991057; doi:10.7554/eLife.85324)
Supplement: Figure 5—source data 2. [file elife-85324-fig5-data2.docx]

**1. mmu-mir-101b**

**sequence：**

CTGCTTACTGGCTTATCGAAATTAATACGACTCACTATAGGGAGACCCAAGCTGGCTAGCGTTTAAACGGGCCCTCTAGACTCGAGTGCATGTATGAGCGTGTACTCATGTATACACACTTACACGCTCACAGACTTAAGCTAGCTGTTTGGTCAGCGAGGGGTGGTAAGTGGGAGGGGCAGGTAGATCTGAGACTGAACTGCCCTTTTTCGGTTATCATGGTACCGATGCTGTAGCTCTGAAAGGTACAGTACTGTGATAGCTGAAGAATGGCGGTGCCATCACGTTGAGGGGAGGCGCCTGAAGGATTCTTTTACTTTTTAGAGTTGGCAGTGGGAGGTTTTTGGAACAGAAGATGAAAGGGAATATACTCTCTTAGCAGCGGTACCAAGCTTAAGTTTAAACCGCTGATCAGCCTCGACTGTGCCTTCTAGTTGCCAGCCATCTGTTGTTTGCCCCTCCCCCGTGCCTTCCTTGACCCTGGAAGGTGCCACTCCCACTGTCCTTTCCTAATAAAATGAGGAAATTGCATCGCATTGTCTGAGTAGGTGTCATTCTA

**2. mmu-mir-27a**

**sequence：**

GAGCTCTCTGGCTAACTAGAGAACCCACTGCTTACTGGCTTATCGAAATTAATACGACTCACTATAGGGAGACCCAAGCTGGCTAGCGTTTAAACGGGCCCTCTAGACTCGAGCCAACTGACCCTGTGCTCTGCCTTGGGGGCTCCTGTCGCCAAGGATGTCTGTCTTGGGTACTGGGTAACAGAGAAGCCTATCATGACAACTGGCCTGAGGAGCAGGGCTTAGCTGCTTGTGAGCAAGGTCCACAGCAAAGTCGTGTTCACAGTGGCTAAGTTCCGCCCCCTGGACCCCATCTCCTCAGGCCGCTGCTGCCCACCCTGCTCTGCCTCCTGGCTGGCAGGGCTCTGCCTCTCTCCGGGCTCCGCCGGTACCAAGCTTAAGTTTAAACCGCTGATCAGCCTCGACTGTGCCTTCTAGTTGCCAGCCATCTGTTGTTTGCCCCTCCCCCGTGCCTTCCTTGACCCTGGAAGGTGCCACTCCCACTGTCCTTTCCTAATAAAATGAGGAAATTGCATCGCATTGTCTG

**3. mmu-mir-129-1**

**sequence：**

GAGCTCTCTGGCTAACTAGAGAACCCACTGCTTACTGGCTTATCGAAATTAATACGACTCACTATAGGGAGACCCAAGCTGGCTAGCGTTTAAACGGGCCCTCTAGACTCGAGTCCAGGTCCCAGCTCTGCTTCCCTGACCCCATCTGCAGTCGCAAGCAAGCCCCAGCCCTGGAGGGGCTGCACCCTGCAACCTTCCCACAGCTGTCTCCTTTGGATCTTTTTGCGGTCTGGGCTTGCTGTTCTCTCGACAGTAGTCAGGAAGCCCTTACCCCAAAAAGTATCTACGGGAGGCTTGGTCTACAGGGGAGACCCCCAAGGGCTCCAGGTGAGTCACACCAAACTCAAGACTACCATGTGGATCCAGGGCGTCTTTGCCATACCTTAGGTACCAAGCTTAAGTTTAAACCGCTGATCAGCCTCGACTGTGCCTTCTAGTTGCCAGCCATCTGTTGTTTGCCCCTCCCCCGTGCCTTCCTTGACCCTGGAAGGTGCCACTCCCACTGTCCTTTCCTAATAAAATGAGGAAATTGCATCGCATTGTCTGAGTAGGTGTCATTCTATTCTGGGGGGTGGGGTGGGGCAGGACAGCAAGGGGGAGGATTGGG

**4. mmu-mir-16-1**

**sequence：**

GCTTACTGGCTTATCGAAATTAATACGACTCACTATAGGGAGACCCAAGCTGGCTAGCGTTTAAACGGGCCCTCTAGACTCGAGTGTTGAAAAGGTGCAGGCCATACTGTGCTGCCTCAAAATACAAGGACCTGATCTTCTGAAGAGAGTACCTGTCTTTTTATTCATAGCTCCTATGATAGCAATGTCAGCGGTGCCTTAGCAGCACGTAAATATTGGCGTTAAGATTCTGAAATTACCTCCAGTATTGACTGTGCTGCTGAAGTAAGGTTGGCAATACTCTACAACTGTAATTCAATGTGTATTGATGTTACTTGTGTGTTTACACGGTTTTTCTAATTGCATTAGTGATTTTATGTTTGGTATGATTCTGTAATGGTACCAAGCTTAAGTTTAAACCGCTGATCAGCCTCGACTGTGCCTTCTAGTTGCCAGCCATCTGTTGTTTGCCCCTCCCCCGTGCCTTCCTTGACCCTGGAAGGTGCCACTCCCACTGTCCTTTCCTAATAAAATGAGGAAATTGCATCGCATTGTCTGAGTAGGTGTCATTCTATTCTGGGGGGTGGGGTGGGGCAGGACAGCAAGGGGGAGGATTGGGAAGACAATAGCAGGCATGCTGGGGATGCGGTGG

**6. Arid1a(NM_001080819-3utr(peak))**

**sequence：**

CAAGAAGGGCGGAAAGATCGCCGTGTAATTCTAGACAGCCGTGGGACACCTCCCCTCCCCGTGTGTGTGTGAGTGTGTGGAGAACTTAGAAACTGACTGTTGCCCTTTATTTATGCAAAACCACCTCAGAATCCAGTTTACCCTGTGCTGTCCAGCTTCTCCCTTGGGAAAGCCTCTCCTGTTCTCTCTCCTCCCCACCCTCACTCCCTCACACCTTTCTGTTCCCCATCCTCACCTGCTTCCCTCAGGACCCCACCCTATTTGAAAAGACAAAGCTCTGCCTACATAGAAGACTTTTTTATTTTAACCAAAGTTACTGTTGTTTACAGTGAGTTTGGGGAAAAAAATGGCTTTCCCAGTCCTTGCATCAACGGGATGCCACATTTCATAACTGTTTTTAATGGTTAAAAAAAAAAAAAAAAAAAGGAAAAAAAATACAAAAAAACCCTGAAGGACAAAGGTGACTGCTGAGCTGTGTGGTTTGTCGCTGTCCATTCACAATCTCGCAGGAGCCGAGAAGTTCGCAGTTGTGAGCAGACCCTGTTCACTGGAGAGGCCTGTGCAGTAGAGTGTAGATCCTTTCATGTACTGTACTGTACACCTGATACTGTAAACATACTGTAATAATAATGTCTCACATGGAAACGAGAGAAGACGCTGGGTCAGCAGCAAGCTGTAGTTTTTAAAAATGTTTTTAGTTAAATGTTGAGGAGAAAAAAAATGGCTTTCCCCCCAAAGTATCCTGTGTGAACCTACAACGCCCTGACCTCTTTCTCTCCTCCTTGATTGTATGAATAGCCCTGAGATCACCTCTTAGACCTGGTTTTAACCTTTAGCTGCAGCGGCTGCGCTGCCACGTGTGTATATATATGATGTTGTACATTGCACATACCCTTGAATCTCCACAGTTTGGTCCCCTTCCCAGCTACCCCTTTATAGTATGGCGAGTTAACAAGTTGGTGACCTGCACAAAGCGAGACACAGCTATTTAATCTCTTGCCAGACATTGCCCCTCTTGGTGCAGTGCTCTACAGGTCTCTGTAAAAAGCCCTTGCTGTCTCAGCAGCCAATCAACTTACAGTTTATTTTTTTCTGGGTTTTTGTTTTGTTTTGTTTCATTTCTAATCGAGGTGTGAAAAAGTTCTAGGTTCAGTTGAAGTTCCTGATGAAGAAACACAATTGAGATTTTTTCAGTGATAAAATCTGCATATTTGTATTTCAACAATGTAGCTAAAAACTTGATGTAAATTCCTCCTTTTTTTTCCTTTTTTGGCTTAATGAATATCATTTATTCATCTAGAGTCGGGGCGGCCGGCCGCTTCGAGCAGACATGATAAGATACATTGATGAGTTTGGACAAACCACAACTAGAATGCAGT

**7. Grin2d(ENSMUST00000002848-3utr(peak))**

**sequence：**

CCAAGAAGGGCGGAAAGATCGCCGTGTAATTCTAGACGCGGCTCGGGCCCCCCACCCCCGCCGCCCCCTCGGTCAGCGCAGGCCACGACCCGAGGGGACGCGCTAAGTGGACAAGACCCTCATGGGTTGGGAAGGACAGAAACGGACCTGGCTGGACCTCGCCTGGAGCAGCGTCCTGCGCCCCACCCCCTTGCCTTGGGGGAACTATGAGCCAGAGGGGACTCGGTCGCCCAAATCTCACCCATCCTGAGAGCAAGGGGGCGGGGTTCTTGGAGCCCACCGACTCTTTTTTAAACCCGAAAAGGGCTTTTTAACGTCACCTGATTGGCGGGAGGTGTGGGGGGGTCACGCCGCGCCCCACCCCTACGCTCCGGGCCATGGCCCTCACATCACTGTGCAGCTCCCTAGCCCCAGACACACCTCTAGGGGAGCCTGTTTTTAACCTTTCATTCATCAGGACTCAAAACTGTGAGACGCGTATGCCAAACTGTGACTCCAGACCTTGGCCCCGCCACACAGAAACCCCTGACCCCAGACTGTGACTTCCAACACCTAAAATGATCATCTGACCCCAGACTGTGCCCCGACCAGACTGTGACCCTTCATGTCAGACCTCAATGACCCCTCCCTTTTAGATCCTTGTTGGCTCTGACCTATGACCTCTCCTCCACGAAAGTCCTACCTTTACCTGTCTCCCCCCACTCACCTACCACCCCAGATGCCACCAATCACATCTTCAAGTCCAGCCCTTATTTGTGACCCTTCAGTGATAATCCAGCAGACACTCCACCCCCAGGTCTCCTTTCCCAGATTTCAGCAGGGTCTGAGGCTGGGCTGAGAGGAGGGTCTGGGAGCCCACACCTGACCTTTCCTCCCCTCTCCCGCTCCTACACTGCAGTGTTTGGAATAAAACCATGTTTTTACGCCTCCTCATCTAGAGTCGGGGCGGCCGGCCGCTTCGAGCAGACATGATAAGATACATTGATGAGTTTGGACAAACCACAACTAGAATGCAGTGAAAAAAATGCTTTATTTGTGAAATTTGTGATGCTATTGCTTTATTTGTAACCATTATAAG

**8. Rbfox3(NM_001024931-3utr(peak))**

**sequence：**

GGCCAAGAAGGGCGGAAAGATCGCCGTGTAATTCTAGAAACCTTCCACCGTCTCCTTCTCGGACCATGAAGGGCAAAAACAAAAAAACAAAAAAAATCACAAAACAAAAAACAAAACAAAAAAAGATGTTAAGATCCAAGCAACAACAGCAACAACAACAAATACCAACCAAACCAAGAGGCATCCAACCAAGTCCAAGTCCACGTCTGGCGTACACCCGCATCGAGGGAGCTCGTCCCCCAGGGGGCGCCGGAGAGTGGCTTTGCCTGGCTGGCGGTGTAGTGGGGACGCGGCCAGACACTGGGGAAGACCTGGAGGGGGAACGTGGCCAGTGGGTGTGGGGCGGGGGGCTCCGGTATCTATCCAGTCTTCCTTTCCCACCCTGCTCCCCAGACAGGGGGTTCCTAGGGTGGACCAGGCCCCTGAGAAGGAGCGGCCAGCTGACTTGGGCTATTGCCTGCTGAGCCCTGAAGGGGCTGGAGCAGTTAGGGGCCAGCGGCTTATCACTCATCCCAGGTCTGCAGCCTGGCCTCATTGAATGTTGCTGCCAGTGCCGTCAGCTATCCAACCAGCCGCCCTGGGTTCCAACTCCCAATGCAAAGGGCTGACACGGCTCCAGAACTCTGGGGCACCCCATGCTCCGCTCCCCTATGTGAGAAAGCTTCCGGCTACAAAGAACAAAGAAAGCCCAGCCAGCGAGGACCCAGTCCTCACGCTGTGAACGTGGACAGGTGGGCCCTCGGGCACAGGGTTGTTTGTCGTGGCAGCCTCCCTGAAGGAGGCTCCGTGGGCTTGAAGGCCAGCCTCAGCTGTCTTCTAGGACCCAGCCCTCCTTTCCACTCCCTTCTGTAGGCTCCGTTCTGCTGGGGAGTGGGGATTGAGACGGGCCAGCCCTGGGCTGCACCTAGATGTTGGGAGTCAAGGTGCTGTGGGTCTGGAGGGCCGCAGTCAGAACAGGACCCATGGGAAAGGCAAGGGATGGGGAAGGTCTGGGCTGGCCCTGCCACGGGGAAGGCCGGCTGGGCTGCAGCCCGTTTGCTCCCTGGGCAGGTAAAGTGTGGGAGTCACGGGAAAGCAGCAGGCCCAGTGGATGGAACAGGGAAGAGGTATCAAGAAGCCCCTGGGCAAGGCAGGGATAGGTGGAGTAGGGTTAACAGCAAAAAAGAAAAAAAAAAAAAAATTAACAGAAACCAAGGCTCCCGACGCGGCACCACTGTCTCATGACACCTCAAAATAAATCACCTCACTTTCTATCCAGCATCAGCCACAGAAGCTATGGAAACGGAACCCTCCTTTTCTATTCCTGATGTACATAGCCCCGCGCCTGCCTCTGGCTCTGTCCTCTGTACGGAGCCCCTTGCCTTCTGCTGTTTCGGACCCTTTTCCCGCAGCCCCTCCCAGGACTGCAGCCGAGCCCCAGGCTTCCTTTCTTACCATTCTGTATGCTTCCACGGTGTGACCATTCAAATTAACAGTATTATTATTAAGATTAATAAAGATTTCTTTCTTCAAACCAGTCTAGAGTCGGGGCGGCCGGCCGCTTCGAGCAGACATGATAAGATACATTGATGAGTTTGGACAAACCACAACTA

**9. Wdtc1(ENSMUST00000043305-3utr(peak))**

**sequence：**

AGATCGTGGATTACGTCGCCAGTCAAGTAACAACCGCGAAAAAGTTGCGCGGAGGAGTTGTGTTTGTGGACGAAGTACCGAAAGGTCTTACCGGAAAACTCGACGCAAGAAAAATCAGAGAGATCCTCATAAAGGCCAAGAAGGGCGGAAAGATCGCCGTGTAATTCTAGAACCCTCCTGGCCCCAGTACCCAGCCCTGTGCAGGAGATCAGAGGGCTGGGCTCTGTTTTGGTTTGTCTTTCCCACCCCCATTTTTCATTCTCCCCCCCCCCCTGTTTTGTTTGTTAGTTTGACTTTGTGGGTGGGGATTGCTGCAACTTGCTGGCTTTCTGACTCTCGGGCTGATTGTCCCTTGACTGTCCCCAGCCCCAGCAGAAAAGTAGGAGGGGAAGCCCCTCCGTATGCCCTTACCTCCTGCTTCGGTGTCTCTGGAAGGCTCTGGCCCTTCTCAAAATCCTTCAGCCTCCAGTGGAGAGGCAAGAGCTGAAGCTGTCTTTACGGACTGCCCAGCCCCTCCGCGGGCAACAGAAAGGGAGTGGAACTCCCTGCTGGCCGAGGCTTGCCTGGCCTGTTGTTGCCACTCTCCTGACTCCCGGGCTAGCTCCCCTGTGGTCTTTCCGGAAGGTCCCTCCACAGCCACCAGTGTTCGGGTGGAAGCATAGGAAACAGGATCCCGCAGTGGCAAGGTCAGGCCACCTGCCTGGGGGCAGTGGAGCTTCCTCCCCCGCTCCTGGCCTGTCCTAGGCTGTCTCGCTGTCTCTGAGGGCCTTGCCTTTCCTACAGTGTGACGGGGTGGGGTCCCTCCCTCCCCACCCACCTGCCCATTCGAGTCCTTACGCGCCATGGCCGCTCTCCTGGGGCGCTTCTCGGCCTCACTGTGACCTTTCTGCCAGAGCTCCCCGCCAGGCCTACCCTGCTGAGGTGGCTCTTCCTAAGGACCCTTTGCACCCTCTCCGCCCTCCTTCCCACCCACATGCTGAGCCGCCACAAAGACCAAAGAAGTGATGGCTTTTCTCTGTCCCCTGCTGCTCTGGGGGGAGGGGTTGGGAAAGTCCTTAGCTCAGCCATCCCCCCTCAGCAGCCCCGAGCTTTACACTGGACACAGCGGCCACTCCACCACCAAGCCTCCCTCTCCCTCTGGCCCCTTGGTTCCTGCTCTGGAGCCGGGAGTTGGTTCATGCCAGCCTCCCCCAGCCCTGTGTGCTTCCTCTGGGCGGCAGCCCTCCAGCTGCAGCCTCTGGGGAGAAGCAGCCTCCCTTCCCTCCCTCCCTCCATCCCTCCCTCTCTCTCCTGTCCTCTCTCCCTCCCTCACCCCTGTCTCTGCCAGGTGCCTCCTCTCAGTCTAGCTTCTGAGCAGCCCTGGAGACTGGAGGGCCATGTTAAAAGCTTTTTCACAGTTTTAAGGAGACGAGGGGGGTGAGGCTAGTGGTGGGGGGTCTGGCACCATCTCATTGCTTTAATCTCAGCGACACAGGTGGCAGCTTCTCCCCCTTCCTGCCCACCCCAGGTCCTCTTCCCACCCTCTCTCCTAGCTTGGTAATGAAGTGTATTTATTGGTGAAGGAAACAGCTGCTCTGCTGCTTCTCCTGCTGCTGGGACTTGCTCCCCTGTCTCTTCCTCATGACCTTTCTCAAGCCAGGGAGGGGTAGCGAGAGTGTTGAGCTGGGCCCTGGGGCACAAGAACTAGCCAGGGTCCCTTTGCTTTCCTGTGTTCAAGCCACCTGCCATCCCCCCTGTCCATCCTCCTGTCTCCCGCCCGCCTGCCTAGCCTGGACCCTGCAGTGTGGAGAGACACATAAGCCTTACTGTCCTCTGGGGCAGGAGCCGAGCCTTTTGTTGCTCTGCTCCCAGGAGAGTGAGGGTGACACAATTGATTAAAACCATTTTGTTCTAGCTGTGATCTAGAGTCGGGGCGGCCGGCCGCTTCGAGCAGACATGATAAGATACATTGATGAGTTTGGACAAACCACAACTAGAA

**11.Trmt6(NM_175113)**

**sequence：**

CCAACTGCACCTCGGTTCTGCTAGCGTTTAAACGGGCCCTCTAGACGCCACCATGGAGGCATCAGCTGCGGAGCAGCCGAGCTCACCGCCCCCGCCTCTCGGGGACCACTGCATCCATGACGGTGACTTCGTGGTGCTGAAGCGAGAAGATGTGTTCAAAGCAGTGCAAGTCCAGCGGAGAAAAAAAGTAACTTTTGAAAAGCAGTGGTTCTACCTGGATAACGCCATCGGCCATAGTTACGGCTCAGCGTTTGACGTGAGCAGTGGAGGCAGCCTTCAGCTCAGGAAGAAGCTGGAGGAGCCCGCGTCAGAGACCAAAGAAGCAGGCACTGATAATCGAAATATAGTTGATGATGGAAAGTCCCAGAAACTTACTCAAGATGATATAAAAGCTCTGAAAGACAAGGGCATTAAAGGAGAGGAAATAGTTCAGCAGCTAATTGAAAATAGTACAACATTCCGTGACAAGACAGAATTTGCCCAAGATAAATATATTAAAAAGAAGAAGAAAAAATATGAAGCCATCGTTACTATCTTGAAGCCATCTACCCGTATTCTTTCAATTATGTATTATGCAAGAGAACCTGGAAAAATTAACCACATGAGATATGATACACTAGCCCAGATGTTGACGTTGGGAAATATCCGTGCCGGCAATAAAATGATTGTCATGGAAACGTGCTCGGGCTTGGTGCTAGGTGCCATGATGGAACGAATGGGAGGCTTTGGCTCCATTATTCAGCTGTATCCTGGAGATGGACCCGTTCGGGCGGCAACAGCATGTTTTGGATTTCCCAAATCTTTCCTCAGTGGTCTTTACGAGTTCCCTCTCAACAAAGTAAACAGTCTCCTCAATGGGACATTTTCTGCTGAGATGCTGTCCTCAGAGCCTAAAGACAGCACTCCAGTTGAAGAAAGTAACGGTGAGCTTGAGGAGAAAGAGATTGCTGAACAAGCGGATGAAGACAACATTGTGGATGCCGCAGAAAACAACTCAGGAGAACAGAGACCAATGGAGATTGTTCCTGGGGACCCAGAGAATAAGGAGCCCAAAGAAAAAAGAAGCAAAAGAGATTATATTCAGGAAAAGCAAAGGAGACAAGAAGAGCAGAGGAAAAGACATCTGGAGGCTGCTGCTCTGCTGGGAGAAAGAAATGCAGATGGTTTGATTGTGGCCAGTCGTTTCCACCCCACGCCCCTGCTGCTGTCTTTGCTGGACTTTGTAGCCCCGTCAAGGCCGTTTGTGGTCTACTGTCAGTATAAAGAGCCTTTGTTGGAATGCTACACAAAACTTCGGGAGAGGGGAGGAGTCATTAACCTGAGGTTGTCTGAAACCTGGCTCAGAAATTACCAGGTTCTGCCAGATCGGAGTCATCCCAAATTATTGATGAGTGGCGGTGGAGGGTACCTTCTGTCAGGGTTCACTGTTGTCTCAGACAGCCTTCGGGCAGACCCCAGCCTCAAGTCCTGCACAGGCGCTTTAGACCCACACAAGGCTGAGGAGCCAGCAGCTAAAAAACAGAAATGCATGGAATCTGCCTCTTAATCTAGATAGTTAAACCGCTGATCAGCCTCGACTGTGCCTTCTAGTTGCCAGCCATCTGTTGTTTGCCCCTCCCCCGTGCCTTCCTTGACCCTGGAAGGTGCCACTCCCACTGTCCTTTCCTAATAAAATGAGGAAATTGCATCGCATTGTCTGAGTAGGTGTCATTCTATTCTGGG
